# Tracheobronchomalacia in a patient on invasive mechanical ventilation: the role of electrical impedance tomography in its detection and positive end-expiratory pressure titration

**DOI:** 10.1590/S1806-37132015000004410

**Published:** 2015

**Authors:** Olívia Meira Dias, Eduardo Leite Vieira Costa, Daniel Antunes Silva Pereira, Caroline Nappi Chaves, Samia Zahi Rached, Carmen Silvia Valente Barbas

**Affiliations:** 1 Attending Physician. Department of Cardiorespiratory Diseases, Heart Institute, University of São Paulo, School of Medicine, Hospital das Clínicas, São Paulo, Brazil; 2 Attending Physician. Respiratory Intensive Care Unit, University of São Paulo, School of Medicine, Hospital das Clínicas, São Paulo, Brazil; 3 Attending Physician. Department of Cardiorespiratory Diseases, Heart Institute, University of São Paulo, School of Medicine, Hospital das Clínicas, São Paulo, Brazil

## To the Editor:

Tracheobronchomalacia (TBM) is a disorder caused by weakness of the tracheal and bronchial walls, together with softening of the supporting cartilage, resulting in excessive expiratory collapse. ^(^ 1 ^)^ Although some individuals with TBM are asymptomatic, others present with symptoms such as dyspnea, hemoptysis, wheezing, and chronic cough.^(^ 1 ^-^ 3 ^)^ Because the symptoms are nonspecific, TBM can be easily overlooked or misdiagnosed as other obstructive airway diseases, including asthma and COPD.^(^ 4 ^)^

In TBM patients with acute respiratory failure, noninvasive ventilation is a therapeutic option, because positive end-expiratory pressure (PEEP) can prevent airway collapse.^(^ 5 ^-^ 7 ^)^ Kandaswamy et al.^(^ 8 ^)^ reported that, among patients with respiratory distress who failed weaning from mechanical ventilation or required reintubation in the ICU, the prevalence of TBM, identified on CT scans of the chest acquired only days before intubation, was 1.6%. However, to our knowledge, there have been no reports of TBM being diagnosed during invasive mechanical ventilation.

Electrical impedance tomography (EIT) is a noninvasive, radiation-free monitoring tool that provides real-time imaging of ventilation at the bedside. Here, we report a case in which the combination of CT and EIT scans of the chest allowed us to make the diagnosis of TBM and to determine the best PEEP titration for preventing airway collapse in an intubated patient.

A 66-year-old female was admitted to the emergency room complaining of breathlessness. She had a history of recurrent episodes of wheezing and dry cough, both of which partially improved after treatment with aminophylline and inhaled short-acting bronchodilators. Her past medical history included orotracheal intubation, for severe bronchospasm, five years prior. She reported no fever, sputum production, or other symptoms. She stated that she had not been exposed to any inhaled allergens, had no known allergies, and had no family history of asthma. She reported that she was not a tobacco user but had long been exposed to biomass smoke from cooking.

On physical examination, she was in respiratory distress, presenting with accessory muscle use, her RR was 28 breaths/min, and her SpO_2_ was 96% while breathing room air. Examination of the lungs revealed prolonged expiration and diffuse wheezing.

The results of the radiographic assessment and laboratory exams were unremarkable. Partial symptomatic relief was achieved after inhalation therapy with ipratropium bromide and fenoterol, together with intravenous hydrocortisone. She was discharged home but returned to the emergency room with bronchospasm minutes later. Although she was then treated with additional doses of inhaled bronchodilators, as well as intravenous magnesium sulfate, her dyspnea worsened. A few hours later, she became comatose, requiring orotracheal intubation and admission to the ICU.

**Figure 1 -:**
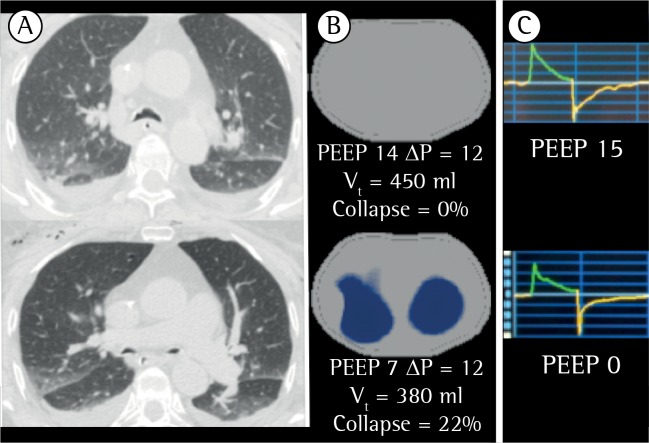
In A, an HRCT scans of the chest showing bilateral small pleural effusions with partial lower lobe atelectasis, and a reduction in the cross-sectional diameters of the trachea and main bronchi, mainly in the subsegmental divisions of the lower lobes, bilaterally; in B, electrical impedance tomography scans showing that the use of a higher PEEP prevented airway collapse an expiratory flow curve indicative of airway obstruction; in C, an expiratory flow curve indicative of airway obstruction and a dramatically improved expiratory flow curve, no longer indicating flow limitation, after an increase in the positive end-expiratory pressure (PEEP).

At ICU admission, the patient was deeply sedated with fentanyl and midazolam, was started on inhaled albuterol (400 μg/h), received intravenous hydrocortisone (200 mg every 6 h), and was started on continuous cisatracurium infusion. Respiratory mechanics were measured during volume-controlled ventilation; the PEEP was set at 5 cmH_2_O; the FiO_2_ was set at 40%; the RR was 12 breaths/min; the flow rate was set at 60 L/min; the inspiratory pause duration on plateau pressure was 2 s; and the tidal volume (10 mL/kg) revealed low lung compliance (26 mL/cmH_2_O), high airway resistance (24 cmH_2_O/L/s), and an intrinsic PEEP of 8 cmH_2_O. The expiratory flow curve was indicative of airway obstruction (Figure 1). Chest X-rays performed 24 and 48 h after ICU admission showed atelectasis of the left lower lobe and of the right lower lobe, respectively, without any differences between the two time points, in terms of the ventilatory parameters.

At 48 h after ICU admission, an HRCT scan of the chest showed bilateral small pleural effusions with partial lower lobe atelectasis, and a reduction in the cross-sectional diameters of the trachea and main bronchi, mainly in the subsegmental divisions of the lower lobes, bilaterally, all of which is consistent with a diagnosis of TBM (Figure 1). On the basis of that working hypothesis, the patient was maintained on pressure-controlled ventilation and the PEEP level was changed from 5 cmH_2_O to 15 cmH_2_O, all other parameters remaining unchanged. The wheezing on auscultation suddenly disappeared, tidal volume increased from 227 mL to 398 mL, and the shape of the expiratory flow curve dramatically improved, no longer indicating flow limitation (Figure 1).

In order to choose the PEEP level that would represent the best compromise-maintaining airway patency while avoiding lung overdistension-we titrated PEEP with EIT (Table 1). After a lung recruitment maneuver, we kept a constant driving pressure of 15 cmH_2_O and decreased the PEEP level by 2 cmH_2_O every 2 min until we reached a PEEP of zero. The PEEP level that allowed minimal lung overdistension and prevented airway collapse was 12 cmH_2_O (Figures 1D and 1E).

**Table 1 -:** Titration of positive end-expiratory pressure and the corresponding tidal volumes.

PEEP (cmH_2_O)	V_T_ (mL)
13	450
11	440
9	420
7	380
5	327
3	230
ZEEP	144

PEEP: positive end-expiratory pressure; VT: tidal volume; and ZEEP: zero end-expiratory pressure.

The patient showed considerable improvement in her ventilation parameters. However, because of difficult weaning, she was tracheostomized at discharge.

The conventional approach to detecting TBM is by fluoroscopy or through direct observation on bronchoscopy, in which a narrowing of > 50% in the sagittal diameter of the trachea is considered diagnostic of the disorder.^(^ 4 ^)^ Expiratory CT scans have been validated as a method to evaluate collapse of the airways, a 50% expiratory reduction in the cross-sectional area of the airway lumen being considered diagnostic of TBM in symptomatic individuals.^(^ 4 ^,^ 9 ^-^ 11 ^)^ Although we cannot guarantee that, in the case presented here, the CT scan was obtained at full expiration, the clinical history and CT findings were highly suggestive of TBM. Our patient had also undergone bronchoscopy during a percutaneous tracheotomy performed in the ICU, with sedation and muscle paralysis. However, a detailed evaluation of the respiratory dynamics was not possible, because the patient was not breathing spontaneously.

Previous reports have addressed the importance of noninvasive ventilation to maintaining airway patency, decreasing pulmonary resistance, improving expiratory airflow obstruction, and reducing the inspiratory transpulmonary pressures required to initiate airflow, thus decreasing the work of breathing.^(^ 5 ^-^ 7 ^)^ To our knowledge, this is the first study to address the use of EIT in the ventilatory management of TBM in patients on invasive mechanical ventilation. The respiratory flow curves seen after we increased the PEEP helped show the importance of PEEP as a pneumatic stent to open the previously collapsed airways. In addition, the use of EIT allowed the bedside diagnosis of TBM and the titration of the PEEP. It is noteworthy that EIT was extremely helpful in that it informed decisions regarding changes in the ventilatory strategy, because high PEEP levels are not desirable in patients with obstructive lung disease, who are at a high risk for the development of dynamic hyperinflation. Coincidentally, the PEEP level that was set empirically was similar to that determined to be ideal based on the EIT analysis.

The lability of airway opening and closing in TBM predisposes patients to severe bronchospasm crises that are refractory to conventional therapy. Even for pulmonologists and critical care physicians, the diagnosis of TBM is a challenge. Therefore, it should be included in the differential diagnosis of refractory bronchospasm in ICU patients presenting with predisposing factors for TBM.
